# Trends in the production of scientific data analysis resources

**DOI:** 10.1186/1471-2105-15-S11-S7

**Published:** 2014-10-21

**Authors:** Jason Hennessey, Constantin Georgescu, Jonathan D Wren

**Affiliations:** 1 Computer Science Department, Boston University, 111 Cummington Street, Boston, MA 02215, USA; 2 Arthritis and Clinical Immunology Research Program, Oklahoma Medical Research Foundation, Oklahoma City, OK 73104-5005, USA; 3 University of Oklahoma Health Sciences Center, Department of Biochemistry and Molecular Biology, USA; 4 University of Oklahoma Health Sciences Center, Stephenson Cancer Center, USA; 5 University of Oklahoma Health Sciences Center, Department of Geriatric Medicine, USA

## Abstract

**Background:**

As the amount of scientific data grows, peer-reviewed Scientific Data Analysis Resources (SDARs) such as published software programs, databases and web servers have had a strong impact on the productivity of scientific research. SDARs are typically linked to using an Internet URL, which have been shown to decay in a time-dependent fashion. What is less clear is whether or not SDAR-producing group size or prior experience in SDAR production correlates with SDAR persistence or whether certain institutions or regions account for a disproportionate number of peer-reviewed resources.

**Methods:**

We first quantified the current availability of over 26,000 unique URLs published in MEDLINE abstracts/titles over the past 20 years, then extracted authorship, institutional and ZIP code data. We estimated which URLs were SDARs by using keyword proximity analysis.

**Results:**

We identified 23,820 non-archival URLs produced between 1996 and 2013, out of which 11,977 were classified as SDARs. Production of SDARs as measured with the Gini coefficient is more widely distributed among institutions (.62) and ZIP codes (.65) than scientific research in general, which tends to be disproportionately clustered within elite institutions (.91) and ZIPs (.96). An estimated one percent of institutions produced 68% of published research whereas the top 1% only accounted for 16% of SDARs. Some labs produced many SDARs (maximum detected = 64), but 74% of SDAR-producing authors have only published one SDAR. Interestingly, decayed SDARs have significantly fewer average authors (4.33 +/- 3.06), than available SDARs (4.88 +/- 3.59) (p < 8.32 × 10^-4^). Approximately 3.4% of URLs, as published, contain errors in their entry/format, including DOIs and links to clinical trials registry numbers.

**Conclusion:**

SDAR production is less dependent upon institutional location and resources, and SDAR online persistence does not seem to be a function of infrastructure or expertise. Yet, SDAR team size correlates positively with SDAR accessibility, suggesting a possible sociological factor involved. While a detectable URL entry error rate of 3.4% is relatively low, it raises the question of whether or not this is a general error rate that extends to additional published entities.

## Introduction

Technological advances have enabled the rapid production of data in many scientific fields [1]. Because gathering data is frequently the beginning point for scientific inquisition rather than the end goal, the number of publications that mention the use of software, databases, web servers or informatics components has increased steadily over the past several decades [2]. The field of bioinformatics has grown, in large part, commensurate with this increase in data, and focuses on developing methods to better understand the implications of gathered data. These methods encompass computer programs, databases and other Internet-accessible software products that we will collectively refer to as Scientific Data Analysis Resources (SDARs). Some SDARs have a profound impact on science - three of the four most cited papers in the past 25 years (as of March 26^th^, 2014, according to Web of Science) were SDARs, including BLAST for sequence analysis (cited 37,641 times), Clustal-W for multiple sequence alignment (cited 40,364 times), and SHELX for protein structure determination (cited 35,311 times) [3]. However, one concern with SDARs is that, due to their digital nature, they can suddenly disappear. That is, the equipment hosting the SDAR may become inaccessible for various reasons outside the control of the authors, such as catastrophic data loss, maintenance neglect, or loss of funding to support the resource. Alternatively, new methods may replace old ones, and links to the obsolete methods may simply be retired.

Internet-accessible resources, locatable by their Uniform Resource Locator (URL) addresses, are being increasingly used in scientific publications. However, unlike print, URLs are dynamic and can not only change in their content but become inaccessible. This phenomenon, URL decay, whereby URLs become inaccessible in a time-dependent manner, has been documented in numerous studies to date and has been reported across academic disciplines (e.g., medicine, law, business, social science, etc.) [4-6]. These problems in the persistence of online resources are being dealt with in a variety of ways, such as archival sites like http://webcitation.org[7], and others such as the Neuroscience Information Framework (http://www.neuinfo.org/) [8] to identify, catalog and standardize existing resources.

Here, we are interested in a related phenomenon - factors that are associated with historical SDAR production and whether or not any of them are also correlate with eventual decay. For example, does the number of authors publishing a SDAR correlate with its stability? In terms of vigilance in maintaining the online presence of SDARs, there are several possibilities as to what factors are most effective with reference to the original team creating the SDAR. It's possible that online stability correlates with the number of authors on the paper - perhaps because there might be more people with a vested interest in its maintenance and more people able to maintain it. Or, perhaps more authors might dilute the perception of personal responsibility for SDAR decay, and thus fewer authors per paper might lend itself to greater SDAR stability. What is the distribution in SDAR production by institution and does it differ from scientific research in general? How many groups publish multiple SDARs and are their SDARs more or less likely to be accessible? Alternatively, SDAR decay may be more a function of external factors, such as whether a project to create the SDAR was underfunded, a hosting institution changing policies regarding external access of internal network content, or a lack of interest from the scientific community in using the SDAR.

An interesting corollary to these questions is to contrast SDARs with the biomedical literature in general from an economic perspective. If publications are the currency of academia, can we measure the "health" of its economy? To that end, the Gini coefficient (aka the Gini index or Gini ratio) has been employed as a measure of wealth disparity by economists and sociologists to reflect the contrast between the richest and poorest in a nation. Ranging from 0 (a completely equal distribution of wealth) to 1 (a complete concentration of wealth), it is defined as the coefficient between the area below a Lorenz Curve (the amount of wealth accounted for by a certain percentage of the population) and the area between it and the line of equality (going from 0,0 to 1,1) [9]. By contrasting publication production between biomedical literature and SDARs, we can estimate whether or not SDAR production tends to be a product of those with infrastructure and resources (i.e., wealth in the traditional Gini coefficient model) or is more of a function of individual initiative and effort.

## Methods

All of the extracted data, including MEDLINE URLs, abstracts, author names, dates and institutions, were obtained from the May, 2014 release of the National Library of Medicine's MEDLINE XML dataset [10]. To prevent the introduction of bias created by certain journals appearing in the MEDLINE index sooner than others, the current year (2014) was excluded.

Institution names and their ZIP codes pertaining to individual citations were extracted from the *Affiliation *element, using the "extract_data.py" program, primarily using heuristics and regular expressions. One of the primary goals in extracting the names was to find the most generic identifier that uniquely pertained to an entity so that we could focus on discussing institutions, though this was challenging due to different punctuation, spelling, abbreviations and languages. The *Affiliation *string was first tokenized using commas, after which a prioritized set of keywords were searched within the substrings to identify high-level entities. Due to the noisy nature of the data, all institutions only appearing once were excluded from analysis.

Attempts were made to handle the international and multilingual nature of biomedical publishing. Some keywords were introduced to cover their English equivalents (like "hôpital" (French) and "Istituto" (Italian)), while others only needed to be shortened due to their common lingual history (like "Universi", which matched English, French, German, Italian, Portuguese and Spanish). Unicode text was reduced to its closest English transliteration using the unidecode python package as it was observed that names with non-English characters were inconsistently translated already. All comparisons were done in a case-insensitive manner.

Various functions of standardization were applied (such as eliminating a leading "The"), the most controversial of which would likely be the dropping of any text after "University" if there was text before it. This reduced names such as "Foo University School of Medicine" down to "Foo University" while leaving others like "University of Foo" as they were. This forced judgment calls to be made in terms of what constitutes an institution, like in the case of the most represented institution, the University of California system, which is managed by a central board of regents. At the same time, there are many researchers at universities who list their college (such as a medical school) as their primary entity, making it difficult to fold them back into their university. With over 12 million institute strings, each heuristic added also has the potential to introduce a new parsing error.

ZIP codes were extracted simply by verifying that an entry looked somewhat USA-related (containing "USA" or "United States" or at least not containing another country's name) and looking for a 5 digit number, preferably toward the end of the string.

URLs were extracted from the XML abstracts using a Visual Basic program as described in [6]. To determine their availability, they were then tested over a period of 10 days at 3 random times per day, following the same protocol and using the same "check_urls_web.py" program previously published in [5]. Previous results in [4-6] showed a very small percentage of URLs were only intermittently available (about 3% of URLs were available between 0% and 90% of the time), and the current survey is consistent with that (Additional file 1), with 2% showing intermittent availability. For convenience, we defined a URL to be "inaccessible" if it was accessible less than 3 times of the 30 it was queried, and "accessible" otherwise. We classified URLs as SDARs by key terms that appeared in the abstract with the URL, including "informatics", "algorithm", "software", "web server" and "computer program".

## Results

### Archival site entry errors are infrequent yet potentially problematic

For analysis purposes, we separated out 2,529 websites (S2 in Additional file 1) that were created by organizations to archive static, non-SDAR content (e.g., text, multimedia) from those that appeared to either be more author-initiated or were for organizational archival of SDARs (e.g., http://code.google.com, http://sourceforge.net, etc). This was done on the basis of the top-level domain (TLD). For example, URLs pointing to Digital Object Identifiers (DOIs), http://Webcitation.org, http://ClinicalTrials.gov, and journal-based archive sites were separated out and excluded from the rest of the URLs. These URLs (particularly DOIs) are expected to be more stable due to their long-term organizational support.

Interestingly, of the 666 DOIs detected, 19 (3%) were inaccessible. Although a very low rate, the very idea behind DOIs is to provide a permanent locator. Upon further examination, nine contained apparent spelling/formatting errors, 4 of which could be corrected and were then accessible. The other 5 were missing a critical field standard to DOI formatting, but what the field should have been could not be determined. The remaining 10 may have also had spelling errors but, if so, were non-obvious. Furthermore, this 3% erroneous entry rate seemed to be a general phenomenon, as 85 of the 2,529 archival URLs (3.4%) were inaccessible. Manual inspection of the URLs (as extracted and as written in PubMed) showed that many of them were incorrectly formatted. For example, two links to the archival site http://www.webcitation.org[7] left off the ".org" suffix. The most common errors were to one of the most highly cited web sites, http://clinicaltrials.gov, in attempts to cite the clinical trials number. The proper formatting is http://clinicaltrials.gov/ct2/show/# (where # is the clinical trials registry number), but dozens of publications have misspelled the URL. For example, hyphenating clinical-trials (which does not automatically redirect), misspelling "trials" as "trails", but most often just not correctly formatting the URL. What fraction may be the fault of the authors, the journal publication process or entry into MEDLINE is not known.

### URL decay continues unabated

**Figure 1:**
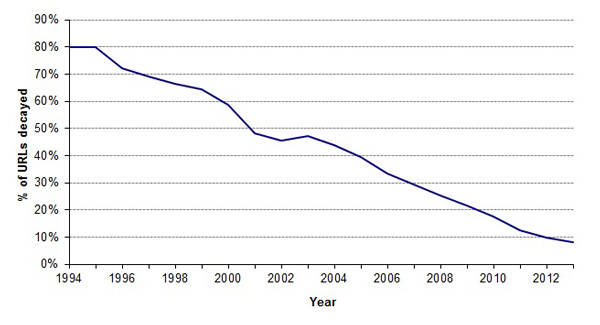
**Fraction of URLs, by year of their publication in MEDLINE, accessible by automated query**. URLs were surveyed daily between March 5th and 14th, 2014.

**Figure 2:**
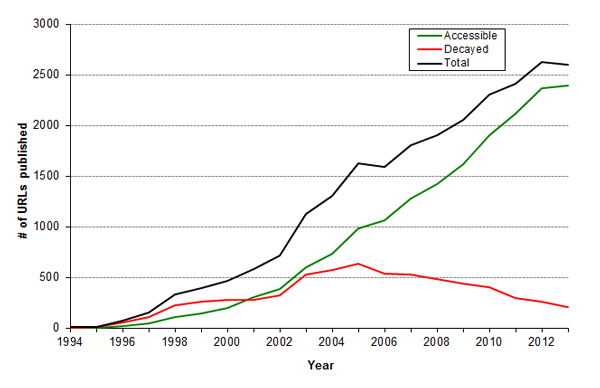
**Number of unique URLs published each year since 1994 - when graphical methods of navigating the Internet became widespread**. Shown are the total number of URLs published during each year, the number of URLs from that year available when surveyed in 2014, and number decayed at the time of the survey.

After excluding archival URLs, there were 23,820 unique URLs found between 1996 and 2013 (full list for all years given in S3 in Additional file 1), and 11,977 of these URLs were estimated to be SDARs on the basis of keywords contained in the abstract. Although this is the largest cross-MEDLINE URL study to date, the results are as expected: A time-dependent decay curve was observed (Figure 1), which is consistent with prior studies [4-6,11]. A total of 74.2% of unique published URLs were determined "accessible" by our automated method, but this total is in large part biased by the relative fraction of recently published URLs (Figure 2). Note that the accessibility survey does not assess content - some URLs may be accessible, yet the original content may have been lost. The fraction of accessible URLs increases to 76.3% when including archival URLs.

#### The number of authors per SDAR is increasing and correlates with future accessibility

**Figure 3:**
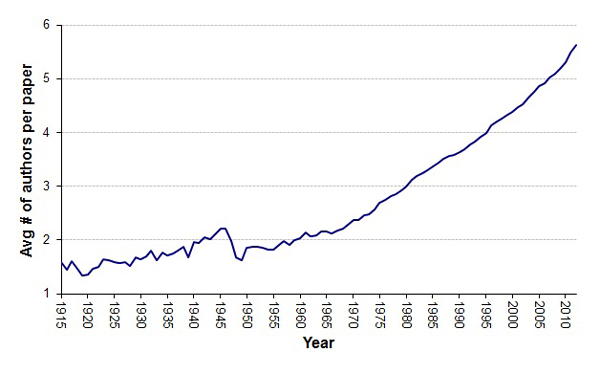
**Growth in the number of authors per MEDLINE paper over time**. Shown is overall growth during the past century.

**Figure 4:**
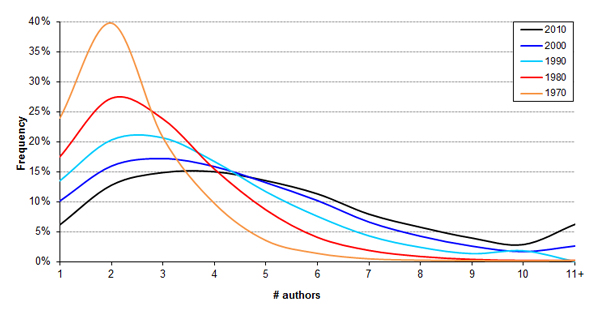
**Histogram of the changing distribution in author number distribution per paper**. Note that the recent apparent acceleration in author number is in part due to an increasing number of "mega papers" with hundreds, even thousands, of authors.

**Figure 5:**
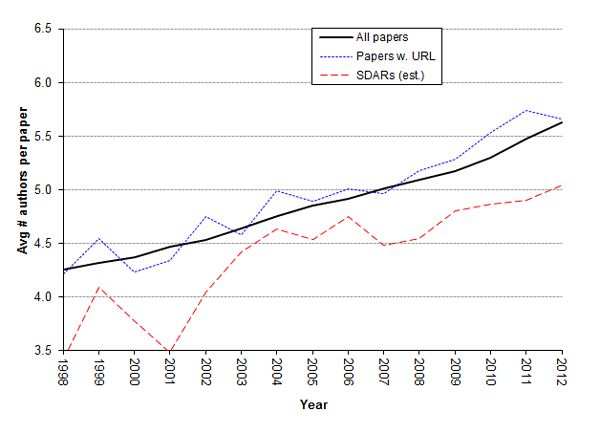
**Comparison in the number of authors per SDAR-producing paper versus overall**.

The average number of authors per paper published in MEDLINE has been increasing steadily and consistently since the 1970s (Figure 3) [12-14]. We assessed in a histogram how authorship frequency is shifting by decade (Figure 4), with a recent increase in the number of papers with more than 10 authors becoming more prominent. The cause of this increase has been debated. One hypothesis is that authorship requirements are becoming more lenient, while others point to the increasing need for specialization in an age of "team science" [15,16]. We examined the authorship trend for SDAR production and find that it is following a similar trend, except the number of authors per SDAR is consistently lower than the average number per scientific publication, as well as lower than the number of average authors per URL-containing paper in general (Figure 5).

**Figure 6:**
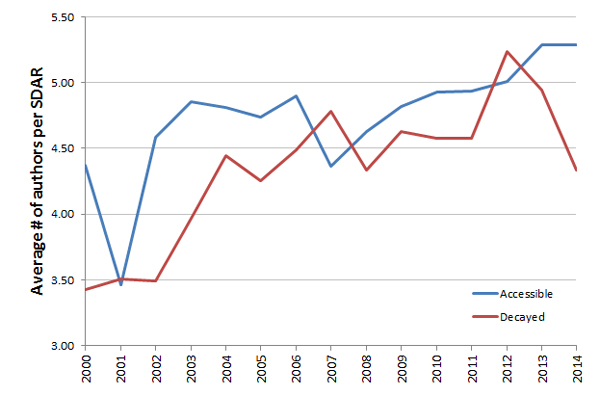
**Current accessibility of SDARs relative to the average number of authors on the original SDAR publication**.

Interestingly, we found that the average number of authors per SDAR paper correlated with the probability the URL was still available (Figure 6). There were 9,708 unique SDARs whose corresponding URL was accessible during this study (avg # of authors = 4.88 +/- 3.59) and 3,332 where the SDAR was not accessible (avg # of authors = 4.33 +/- 3.06). Because the number of authors is increasing with time along with the number of published SDARs, and we furthermore know that availability is a function of time, we used logistic regression to model the probability of SDAR decay as a function of the number of authors, and year of publication. The publication year entered the model with two components: Number of years since publication and SDAR_AGE, a quantitative variable likely to explain most of the decay dependence on year, and the publication year as a factor, with one level for each year, designed to capture the remaining decay dependence on year, not explained by the linear dependence on SDAR_AGE. As shown by Table 1, the ANOVA table of the resulting model, both SDAR_Age (p < 2e-16), and Year (p = 0.022), as well as NumAuthors (p = 0.000879) have significant effects on decay and need to be included in the analysis. Note that the addition of SDAR_AGE in the model, changes the significance p-value of the year variable from 5.49e-295, the p-value in a simplified model with only two predictor variables (see Table 2), to only 0.0225, the value in Table 3. This reinforces that SDAR_AGE, number of years since publication, explains most of the impact of Year on publication decay.

**Table 1:** Logistic regression coefficients from modeling SDAR decay as a function of Number of Authors, Number of years since publication (SDAR_AGE) and Publication Year.

	Estimate	StdError	z value	Pr(>|z|)
(Intercept)	-2.88	0.226	-12.7	3.25E-37
SDAR_Age	0.268	0.022	12.3	6.91E-35
NumAuthors	-0.0236	0.007	-3.34	0.00083
year2004	0.183	0.111	1.65	0.0986
year2005	0.215	0.105	2.05	0.0404
year2006	0.293	0.112	2.61	0.0090
year2007	0.3	0.12	2.51	0.0121
year2008	0.458	0.132	3.47	0.0005
year2009	0.356	0.148	2.41	0.0161
year2010	0.22	0.164	1.34	0.181
year2011	0.23	0.184	1.25	0.211
year2012	0.147	0.206	0.713	0.476
year2013	0.042	0.232	0.181	0.857

**Table 2:** ANOVA table for the logistic linear model decay~NumAuthors+year

	Df	Sum Sq	Mean Sq	F value	Pr(>F)
NumAuthors	1	5.333	5.333	34.269	4.92E-09
year	11	232.047	21.095	135.552	5.49E-295
Residuals	12035	1872.925	0.155		

**Table 3:** ANOVA table for the logistic linear model decay~NumAuthors+year+SDAR_Age

	Df	Sum Sq	Mean Sq	F value	Pr(>F)
SDAR_Age	1	232.419	232.419	1493.474	4.31E-308
NumAuthors	1	1.723	1.723	11.072	0.000879
year	10	3.237	0.323	2.08	0.0225
Residuals	12035	1872.925	0.155		

The resulting statistics for the coefficients of the 3 predictor model, decay ~ NumAuthors+SDAR_Age+Year can be seen in Table 1. As expected, the variable, SDAR_Age, has the strongest influence on decay propensity (p = 6.9e-35). Each additional year passed since publication increases the SDAR decay odds ratio by 1.307 (=e^0.268). The number of authors also had a strong impact on decay evolution (p = 8.32e-04) but in the opposite direction (log odds ratio = -0.0236). That is, more authors on the original SDAR publication correlates with the probability the SDAR will be accessible in the future. The statistics table also shows a slightly higher than expected decay rate in 2008 (p = 5.17e-04, log odds ratio = 0.458) which might account for the remaining marginal significance of the year variable overall (p = 0.0258 as computed by ANOVA procedure). The overall logistic model with three predictor variables has a residual deviance of 11502 on 12035 degrees of freedom. Its improvement relative to the null model, having a deviance of 12890 on 12047 degrees of freedom, is quantified by chi-square statistics of 1388 on 12 degrees of freedom, and a corresponding p-value no larger than 2.2e-16.

#### Decay rates are similar for multi-SDAR and single-SDAR authors

We examined whether or not senior authors that have published multiple SDARs have less overall URL decay (URLs pointing to their SDARs) than those that have published only one. There are competing hypotheses as to whether or not publication of multiple URLs correlates with greater or lesser stability. On one hand, senior authors that produce many SDARs likely have focused on developing the necessary infrastructure and have likely dedicated a substantial portion of their research to providing SDARs. On the other hand, a researcher publishing multiple SDARs might have a single point of failure (e.g., if they change institutions) or simply have too many to effectively keep track of.

We identified 6,600 unique senior author names associated with one or more SDARs. A total of 2,279 of these senior authors had published multiple SDARs, the top 25 of which are shown in Table 4. The average fraction of SDARs still available for multi-SDAR authors was 74% (+/- 33%) vs. 74% (+/- 44%) for single-SDAR authors, but the difference was not statistically significant (p = 0.95, 2-tailed t-test, unequal variance). Raising the threshold to authors that had published five or more SDARs did not change the results. There were 498 authors with 5 or more published SDARs, with a slightly higher average of 76.2% (+/- 25%) accessible, but the difference was not statistically significant (p = 0.089 for the difference between 5+ and single SDAR, 2-tailed t-test with unequal variance).

**Table 4:** Senior authors of papers that report the development of SDARs, including how many SDARs they have published as of May, 2014.

Senior Author	# SDARs
Raghava GP	64
Li Y	30
Casadio R	29
Wang J	28
Noble WS	27
Sonnhammer EL	27
Hamodrakas SJ	27
Wishart DS	26
Gerstein M	25
Bourne PE	22
Dougherty ER	22
Zhang Y	22
Sali A	22
Chou KC	21
Preissner R	21
Giegerich R	19
Lengauer T	18
Altman RB	18
Barillot E	18
Xu Y	18
Ma'ayan A	18
Valencia A	18
Grishin NV	18
Salzberg SL	18
Kim S	18

Only authors with 18 or more are listed here. The author appearing last in the list of authors was presumed to be the senior author of the study (corresponding author is not always listed in the MEDLINE record).

### SDAR production is more widely distributed than research in general

**Figure 7:**
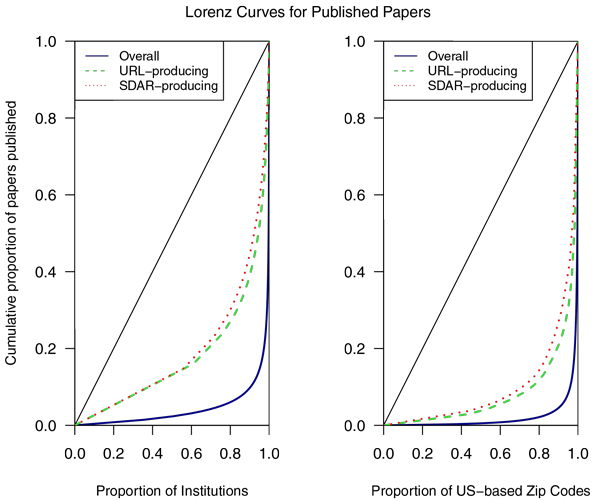
**Lorenz curves comparing the relative concentration of overall biomedical publications produced by different institutions and ZIP codes to those including a URL or SDAR**. The highest-published institutions and ZIPs account for the vast majority of publications overall, however among papers producing SDARs and URLs overall there is markedly less concentration. Perfect distribution is represented by the lines going from (0,0) to (1,1).

Scientific papers tend to be disproportionately produced by a relatively small fraction of institutions, which tends to be a function of infrastructure and resources. We were curious whether or not the distribution in production of SDARs differed from that of publications in general. After parsing out institution names (see methods), 68% of MEDLINE publications came from 1% of institutions, which stands in stark contrast to institutions producing SDARs where the top 1% of paper-producing institutions only produced 16% of SDARs. As a comparison point and as a check on the text processing of institutional data, a geographical comparison using ZIP codes for US-based locations were also extracted and tested, in part because they are simpler and thus more reliable to parse (though ZIP codes can change over time). Similar to the institutional data, we found that the top 1% of ZIP codes produced 71% of publications, while 1% of SDAR-producing ZIP codes accounted for 12% of the total. Illustrating these findings, Figure 7 shows the publication concentration among institutions and ZIP codes with Lorenz curves showing both general publications and SDARs.

Many of the top publication-producing institutions and ZIP codes did not come as a surprise as they are widely known and respected in the biomedical world and can be seen in Tables 5 and 6. Quite interesting, however, is that while these top institutions and ZIP codes are well represented in the SDAR-publishing space, there are notable exceptions. For instance, the University of Manchester and Iowa State University were both among the top 10 SDAR-producing institutions while not placing in the top 50 for overall publications. The European Bioinformatics Institute (EBI) had an even more astounding disparity in ranking, accounting for second place in publishing SDARs while not being in the top 500 in terms of overall publications. Further information on which institutions and ZIP codes contributed the most SDARs can be found in Tables 7 and 8.

**Table 5:** Top institutional sources of scientific publication production.

**Institution**	**# papers**
University of California	148,561
Harvard Medical School	52,156
Johns Hopkins University	44,514
University of Toronto	43,423
Stanford University	41,816
Duke University	40,871
Yale University	40,839
Washington University	39,951

**Table 6:** Top US-based ZIP codes for scientific publication production.

**Zip Code**	**Location**	**# papers**
20892	National Institute of Health, Bethesda, MD	65,484
02115	Boston, MA	56,389
77030	VA Hospital, Houston, TX	53,731
19104	Philadelphia, PA	45,337
48109	University of Michigan, Ann Arbor, MI	36,532
55905	Mayo Clinic, Rochester, MN	33,753
10021	New York, NY	32,380
94143	US San Francisco, San Francisco, CA	30,048
94305	Stanford, CA	28,909
63110	St. Louis, PA	28,268

Locations obtained through US Postal Service website.

**Table 7:** Top institutional sources of SDAR production.

**Institution**	**# of URLs**
University of California	248
European Bioinformatics Institute	90
Stanford University	86
University of Washington	72
University of Manchester	71
Yale University	71
University of Michigan	70
Columbia University	66
Iowa State University	65

**Table 8:** Top sources of URL production by ZIP code.

**Zip Code**	**Location**	**# papers**
02115	Boston, MA	87
94305	Stanford, CA	76
92093	UC San Diego, La Jolla, CA	73
48109	University of Michigan, Ann Arbor, MI	60
50011	Iowa State University, Ames, IA	55
94720	UC Berkeley, Berkeley, CA	54
98195	University of Washington, Seattle, WA	50
90095	UC Los Angeles, Los Angeles, CA	48
20892	National Institute of Health, Bethesda, MD	46
15213	Pittsburg, PA	44

Locations obtained through US Postal Service website.

### Publishing disparities are shown by the Gini coefficient

The Gini coefficient, widely used among social scientists and economists, gives us a useful tool in quantifying the aggregation of publications. For both of the measures we looked at (institution and geography, for which ZIP code was used as a proxy), it was clear that a large proportion of biomedical publications come from a very small percentage of locations. Likewise, while still high relative to most countries, the distribution of URL-producing locations was consistently more distributed than publications overall. Among MEDLINE-publishing institutions, the coefficient stood at .91 while for those producing SDARs it was .62. Compared to institutions, the geographically-based coefficients were both higher, at .96 for all publications and .65 for those producing SDARs. To get a comparative sense of magnitude for the Gini coefficients, it can be helpful to look at contemporary world economies. As of 2014, they range from a low of .25 (Denmark, Japan and Sweden) to a high of .66. The method used for calculating these Gini coefficients was the *Gini() *function in R's ineq package [17]. The coefficient is calculated using the following equation (published in [18]), where n is the size of the population, $x_{1},x_{2},x_{3},\ldots$ is a sorted list enumerating the publication count for different entities (either institutions or ZIP codes) and *µ *is the mean number of publications for all entities:

$$G=\frac{\sum_{i=1}^{n}\left(2i-n-1\right)x_{i}}{n^{2}\mu }$$

**Figure 8:**
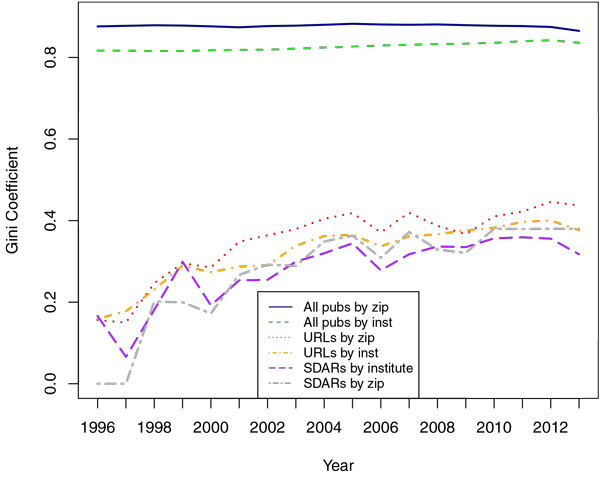
**Per-year Gini coefficient for SDAR and URL-containing papers vs. all publications, broken down by measurement method (ZIP or institution name).** The concentration for SDAR and URL-containing papers appears to be similar, whether measured by ZIP code or institution. So too do the coefficients look similar for publications overall regardless of measurement method.

Given the relatively recent introduction of Internet-based resources into academic publishing, an interesting question to ask is whether their relatively lower concentration when compared to publications overall is a temporary phenomenon. That is, whether SDARs will eventually become more concentrated within a relatively few institutions. To do this, we examined the trajectory of concentration over time by calculating an annual Gini coefficient and looking for a pattern. To that end, four simple linear models were fit for each year from 1996 until 2013 using every combination of location entity (institution or ZIP) and published metric (publications overall or only SDARs). Publications overall appear to show little movement in their annual Gini coefficient, with institutions showing a very slight (significant at 6.6 × 10^-11^) 0.2% increase per year and ZIP codes not having a significant change. SDAR-publishing concentration seems to be on the rise, however, with a 1.2% annual increase among institutions significant at the 3.2 × 10^-5 ^level and 1.9% increase for publishing ZIPs, significant at the 5.8 × 10^-6 ^level. A graph of these values can also be seen in Figure 8.

## Discussion

Programmatic resources for the analysis of scientific data are integral to the modern scientific analysis. Whereas some technologies are still restricted to institutions and researchers with substantial capital, the Internet in combination with a relatively rapid price decline in the cost of computer technology has enabled worldwide access to these Scientific Data Analysis Resources (SDARs). Three of the four most cited papers in all of science within the past 25 years have reported the development of SDARs, which speaks to the extent to which they have influenced research. But the continued accessibility of SDARs remains an issue. We sought here to examine some of the factors related to production and stability of SDARs, such as the size of the scientific laboratories that produce them, a lab's general proclivity to produce SDARs as part of their research focus, and the general distribution of SDAR production among institutions.

We find the average number of authors per accessible SDAR is significantly higher than the average number per inaccessible SDAR. There were no substantial outliers in the data that biased the average (most authors per accessible SDAR was 58, and inaccessible SDAR was 53), so there are several possible explanations. The first is that, perhaps, authors who create SDARs also tend to be users of them and therefore more people tend to have a personal interest in its continued availability. A second possibility may be more sociological in that the more authors per SDAR, the more people there are to contact about its decay, to be vigilant about its accessibility, or to provide options if the primary maintainer becomes unable to maintain it (e.g., changes institutions). It is also possible that larger projects that involve more people also tend to result in a more useful and/or stable end-product.

The distribution of SDAR production suggests that bioinformatics may occupy somewhat of a unique niche among scientific disciplines, as it does not require extensive infrastructure to develop and deploy analysis software. Specialized institutions such as the European Bioinformatics Institute are able to make substantial contributions while not being among the top general players. Consequently, published SDARs can be produced from institutions that lack the resources of more elite institutions. However, whether the SDARs from elite institutions are more stable or more cited is not known and will be the subject of future study.

With a slow but steady annual rise in the Gini coefficient for SDARs, the data may be hinting that this more distributed state of affairs could be gradually approaching the more centralized production of biomedical research. Perhaps nimble early adopters contributed substantially to efforts and now that waning? It may also be that as the novelty of developing and sharing digital analysis resources wears off, large and more established organizations are venturing into that arena, a model that has been suggested for Internet technology in general[19].

Finally, by identifying which URLs should be stable on the basis of their support by organizational entities (e.g., publishers, DOIs, and archival sites like http://Webcitation.org), and should be accessible, we found that a number of published errors have crept into the scientific record, at an approximate rate of 3%. Although this is a relatively low rate, it begs the question of how extensively it permeates reported entities such as numbers (e.g., transposed digits), record identifiers, and names. Variation in URL construction is consistent with other reported variation such as the creation of acronym-definition pairs [20] as well as chemical name spellings within text [21] and even within databases [22], but computers aren't as flexible as humans when it comes to tolerating this type of variation.

There are several limitations to this study. First, we relied upon automated classification of SDARs by keywords present within the abstract. In the future, we plan to crowdsource classification of URLs to better determine which ones are scientific data analysis resources. Another limitation is that not all SDARs are linked to by a URL present within the abstract, so the coverage of published SDARs may be incomplete. For identifying institutions, we attempted to be relatively generic, yet we may have been too generic. For example, the top-publishing institution was extracted as "The University of California", yet this is a system with many different universities. Thus, there is some bias in the institutional results due to the way names were parsed, which is why ZIP codes were analyzed as well. An area of future research would be to identify better mechanisms for identifying institution names, whether through text mining techniques term frequency/inverse document frequency or in combination with a curated list of institutions.

## Conclusion

URL decay continues unabated, but in this study we attempted to analyze a subclass of URL, those reporting the development of Scientific Data Analysis Resources (SDARs). We found average team size for SDAR production tends to be lower than scientific publications in general, although larger team size correlated with SDAR persistence. SDAR production is less dependent upon institutional location and resources, as judged by the Gini coefficient, and groups producing multiple SDARs do not seem to differ in the probability they are still accessible from groups that have produced only one SDAR. Finally, errors are creeping into the public record at a rate of about 3%, rendering their URLs, as written, invalid from the moment of publication.

## Conflict of interest

The authors declare that they have no competing interests.

## Declaration of funding

The authors would like to acknowledge the National Science Foundation (NSF) for funding this research and publication from grant # ACI-1345426.

## Supplementary Material

Additional file 1 **Breakdown in % availability for the URLs queried**. Click here for file
